# Darwinism in Reference to the Eyes of Animals

**Published:** 1872-06

**Authors:** 


### Darwinism in Reference to the Eyes of Animals.

Dr. B. Joy Jeffries made a brief verbal communication on the unity of design in the eyes of man and the lower animals.

In the Harvard University Course of Lectures on the "Anatomy and Physiology of Vision," which I am now delivering at Cambridge, I have had occasion to especially study the unity of design in the visual organs of man and other animals, and by means of my pictures and diagrams I trust to make this evident to the Society. As every illuminated point in nature sends out rays of light in all directions, we have only diverging rays, or those, which so far as the eye is concerned, are practically parallel. There is needed a refractive medium therefore, to bring such rays to a focus on the recipient surface, where the stimulus of light finally causes nerve stimulation, sending the sensation of light through an optic nerve, to the brain or its representative. We may take the human eye as the highest type, and here we have refracting media; namely, the convex cornea and double convex crystalline lens behind it, by means of which diverging or parallel rays of light are focussed on the recipient surface or retina, which lines the interior of the eyeball behind. Thus is formed what exactly corresponds to a *camera obscura*. We have refracting media in whose focus, or in the plane of whose focus, is placed a recipient surface, which recipient surface or membrane or retina contains the means or apparatus for causing the stimulus of light to give rise to a nerve sensation to be transmitted to the brain. Now all eyes, no matter what their external shape or appearance, if they answer these postulates may of course be ranked together as constructed on one design or plan. If we follow down the series of vertebrates we shall find these eyes all formed on this principle of the camera obscura. So also in the *simple* or *additional* eyes of the rest of the animal kingdom, we we shall find a refractive apparatus, in the plane of whose focus is a recipient membrane or retina. And this notwithstanding any difference in shape, size, or general appearance of the vertebrate eye, or the simplicity of these so-called additional eyes of the insects. The only other form of eye existing in the animal series is the *compound* or *facetted* eye of the insects and articulates. The common cornea of this eye is divided up into a large number of distinct facets, (five to thirty thousand), each one corresponding to a tube of pigment, so to speak, in which is found the final termination of the optic nerve fibre. The ray of light which enters through any one of the transparent facets can only affect the optic nerve fibre termination corresponding to it. Hence naturalists and philosophers seemed forced to accord to this form of visual apparatus a different method of perceiving light from that which prevails with the eyes formed on the principle of the camera obscura. Johannes Muller's dictum, more than anything else, seemed to render this an accepted truth. Long ago, however, the strangeness was pointed out of an animal having eyes near each other, whose methods of receiving and perceiving light were on two entirely different plans. Mr. Darwin saw at once this would militate against his theory, and comparatively recent research shows that it is not true. These compound or facetted eyes are also now found to have a refracting medium, in the plane of whose focus is a recipient surface corresponding to a retina. Each one of these facets is in reality a convex lens, and as an old anatomist said, "if we look at a man through these we shall see a whole army of dwarfs." There is, then, a picture formed behind them, just as there is a picture formed on the retina in the vertebrate eye. Moreover, behind each facet there is a refracting body which we will call the vitreous cone and however its shape and appearance may vary in insects and crustaceans, yet its purpose remains the same; namely, that of refracting the light, and together with the convex facet focussing it on the terminal end of the optic nerve fibre behind and in contact with the vitreous cone. Here, then, the stimulus of light produces excitation of a nerve to carry sensation to the brain, or its representative. The facet may represent the human cornea, the refracting vitreous cone next behind it, the crystalline lens, and if we should push back the final optic nerve termini by the interposition of a vitreous humor, the very shape would then resemble the vertebrate type. Thus, we find unity of design in all eyes, vertebrate, simple and compound. The question naturally arises, how can the insect see things singly if thousand of pictures of the same thing are perceived. The answer is that a single fibre supplies many facets, Moreover, eyes seemingly facetted or compound, are on examination, found to be groups of simple eyes close together. No objection has been made to an animal's seeing singly with several simple eyes when these are closely grouped, or to man's single vision with two eyes. A multiplied picture does not go as such to the brain.

Now then, where does light become turned into nerve stimulation. This takes place in the retina, for the optic nerve itself is insensible to light, and where it enters the eyeball is a blind spot in our field of vision. The retina is by no means simply a membranous expansion of nerve substance, but a most complicated structure. Without dwelling upon the arguments in proof I would simply say that its outer layer contains alone the percipient elements, called from their shape the rods and cones. These stand crowded together after the manner of a mosaic, at right angles to the black pigmented surface against which they lie, or rather in which they are bedded. The outer portion of these wonderfully minute little rods and cones is now found to be composed of a pile of plates of a refracting material separated by a less refractive intermediate substance, like a pile of glass plates separated by air. In contact with these come the ultimate fibrillæ of the optic nerve fibres, and in some way the action of the light streaming through this pile of plates stimulates the nerve to a sensation of light to go to the brain. This plated or layered structure of the rods and cones is universal in the vertebrate eye. The portion of the compound or facetted eye which corresponds to the rods and cones is the nerve substance, or its representative, behind the vitreous cone in the pigmented tube, and here also this plate structure has been found in the insects and crustaceans. Thus, then, not only are all eyes so formed as to be adapted to the same laws of light, in having refracting media, in the plane of whose focus a recipient organ turns light into nerve sensation, but the *percipient elements* of this receptive organ, the retina, are also the same, perfectly establishing the unity of design in all visual organs of men and lower animals.—*Proceedings of the Boston Society of Natural History*, 1871.

———:o:———

We make the following extracts from a case reported in the June Number of the *Richmond and Louisville Medical Journal*. Our readers will notice that the tumor was removed by the method of enucleation recommended by us in Ovarian Tumors.

